# Healthcare-associated infections in Italian long-term care facilities: a machine learning analysis of a 12-month cohort

**DOI:** 10.1017/ice.2026.10413

**Published:** 2026-06

**Authors:** Anna Caterina Leucci, Elena Sasdelli, Luana Caselli, Elisa Fabbri, Elena Berti, Costanza Vicentini, Carla Maria Zotti, Katrien Latour, Enrico Ricchizzi

**Affiliations:** 1 Department of Statistical Science, University of Padua, Padua, Italy; 2 Emilia-Romagna Region: Regione Emilia-Romagna, Italy; 3 University of Turin: Universita degli Studi di Torino, Italy; 4 Sciensano, Belgium

## Abstract

**Objectives:**

To estimate the incidence of healthcare-associated infections (HAIs) in Italian long-term care facilities (LTCFs) and to evaluate whether an artificial intelligence (AI) approach, through unsupervised machine learning (ML), could stratify residents into clinically distinct groups with differing susceptibility to HAIs.

**Design:**

Prospective cohort study with 12-month follow-up.

**Setting:**

24 LTCFs in Italy, participating in the European Centre for Disease Prevention and Control 12-month longitudinal study on HAIs in LTCFs, 2022–2023.

**Participants:**

395 residents enrolled across the participating LTCFs.

**Methods:**

Incidence measures of HAIs (rate and ratio) were estimated, using generalized estimating equations. A hierarchical cluster analysis based on residents’ clinical and demographic characteristics was implemented as an unsupervised ML approach.

**Results:**

Overall, 75 HAIs per 100 residents (95% CI, 70.3–78.3) and 0.23 HAIs per 1,000 resident-days (95% CI, 0.11–0.76) were estimated. Respiratory tract infections (29.5%, 95% CI 24.2–31.1), COVID-19 (26.3%, 95% CI 22.1–28.4), and urinary tract infections (15%, 95% CI 11.0–35.4) were the most frequent. Clustering identified two reproducible resident groups: Group 1 (39%), more independent and cognitively preserved, with fewer comorbidities and lower infection incidence; and Group 2 (61%), more dependent and clinically complex, with higher incidence of HAIs. Cluster stability was high (mean ARI = 0.83).

**Conclusions:**

This study confirms the high burden of HAIs in Italian LTCFs and provides exploratory evidence that AI-based clustering can identify reproducible HAI susceptibility profiles in a setting where such approaches have been scarcely applied.

## Introduction

More than 3.5 million Healthcare-Associated Infections (HAIs) are reported in the European Union/European Economic Area (EU/EEA) each year, resulting in more than 90,000 deaths and approximately 2.5 million disability-adjusted life years (DALYs).^1^ The incidence of HAIs with adverse patient outcomes increases with age, making older people more vulnerable to HAIs and their complications.^2^ Long-term care facilities (LTCFs), due to their community-based nature and frail elderly users, are particularly exposed to increased incidences of HAI-associated morbidity and mortality.

The Healthcare associated infections and antimicrobial use in European LTCFs projects, led by the European Centre for Disease Prevention and Control (ECDC), reported a pooled HAI prevalence between 2.6% and 3.7%.^3,4^ Based on national prospective surveillance studies, the incidence of HAIs in European ranges from 2.1 to 11.8 per 1,000 resident days.^5,6^ A pilot Point Prevalence Study conducted in 2022 in Italy, found that 2.5% of residents experienced at least one case of HAI, including COVID-19.^7^ A previous nationwide Italian study estimated that there were approximately 641,065 new HAI cases and 29,375 attributable deaths over a one-year period and the total annual burden was calculated to be 702.5 DALYs per 100,000 inhabitants.^8^ Urinary tract infections (UTIs), respiratory tract infections (RTIs), gastrointestinal tract infections and skin and soft tissues infections are among the most reported HAIs.^9^ A recent European 12-month longitudinal study led by ECDC in 2022–2023 (H4LS study, “Healthcare-associated infections and antimicrobial use in LTCFs—support to a point prevalence survey and a longitudinal study,”)^10^ found a high incidence of HAIs in 65 LTCFs. One in two residents had at least one HAI, leading to hospitalization in 4.3% of total HAIs and death in 4.5% of cases, with RTIs and UTIs accounting for almost half of all HAIs.

LTCF residents are often viewed as a relatively homogeneous, highly frail group with multiple functional deficits. However, an individualized approach recognizes substantial heterogeneity in care needs, particularly regarding cognition and mobility, which are inversely related and major drivers of LTCF admission.^11,12^ The risk of acquiring a HAI, with its associated increased morbidity and mortality, is influenced by specific resident characteristics including advanced age, underlying medical conditions, impaired cognitive and functional status, and the use of invasive devices such as indwelling urinary catheters.^2,3^ In this context, rather than quantifying frailty per se, identifying multidimensional resident profiles based on the concurrent assessment of functional, cognitive, and clinical characteristics associated with differential susceptibility to specific types of HAIs becomes fundamental.

Recent applications of machine learning (ML) models have demonstrated the potential to significantly enhance the prediction of HAIs. Predictive algorithms have been successfully applied to estimate the risk of HAIs in intensive care units,^13^ to identify bloodstream infections at an early stage,^14^ and to recognize patients at risk of acquiring RTIs, including COVID-19, during hospitalization.^15,16^ In many cases, these models have achieved promising predictive performance, with possible applications in supporting clinical decision-making and infection prevention and control (IPC).^17^ However, while artificial intelligence (AI) has already been successfully applied in hospital settings for HAI prediction and antimicrobial stewardship, its use in LTCFs is still largely unexplored.^18^ This study addressed this gap by applying an unsupervised ML approach in on LTCF resident data from the H4LS study.^10^ The aim was to identify resident subgroups with differing risk profiles and HAI susceptibility patterns based on a multidimensional clinical assessment, thereby supporting more targeted and personalized IPC strategies.

## Methods

### Study population and data collection procedures

This research was conducted following the guidelines outlined in the Strengthening the Reporting of Observational Studies in Epidemiology statement,^19^ using data collected within the H4LS study, aimed at assessing the incidence of HAIs and their associated mortality and hospitalizations in European LTCFs.^10^ Informed consent requirements prevented full baseline resident enrollment; therefore, a convenience sampling strategy was adopted rather than a complete census. Eligible residents were those expected to stay in the facilities for at least one year. The population of the present study consisted of 395 residents living in 24 LTCFs (nursing homes, residential homes or mixed type facilities), from two Italian regions, Emilia-Romagna and Piedmont.

The Italian study’s National Survey Coordinator provided each LTCF with the survey protocol and the following operational tools: an Institutional Questionnaire to collect LTCF characteristics, a Resident Questionnaire to collect clinical and demographic information for each resident and a HAI Questionnaire to record each HAI case occurred during the follow-up period. Information on temporary discharges from the facilities were also collected, to include only the HAIs acquired within the LTCFs in the analyses. All data converged into a national study database. The H4LS study procedures are detailed elsewhere.^10^

### Definition of HAI

To ensure unambiguous identification of HAIs, definitions of HAIs followed the McGeer and revised McGeer criteria as adopted in the H4LS study^10^ and described in the ECDC protocol for surveillance in European LTCFs.^20^ No modifications to these definitions were applied in the present analysis. The COVID-19 case definition was based on the positive result of a laboratory test. Asymptomatic cases were not considered. In addition to HAIs acquired within the facilities, HAIs related to a temporary discharge were also included when symptoms occurred more than two days after readmission, since these cases were partially or fully managed and treated within the LTCF.

### Statistical analyses

The following HAI incidence metrics were computed from the crude sample data: (i) percentage, ie the number of HAIs per type, over the total number of HAIs; (ii) ratio, ie the cumulative incidence of HAIs per 100 residents, calculated as the number of HAIs per type divided by the total number of LTCF residents multiplied by 100; (iii) rate, ie the number of HAIs per 1,000 resident days, calculated as the ratio between the total number of HAIs and the sum of follow-up days multiplied by 1,000.

Due to the multi-center nature of the data, Generalized Estimating Equations models were also estimated with an appropriate link function (Poisson or binomial, based on variable types). The intercept of these models was used to obtain a clean measure of the multi-center effects.

Unsupervised ML was used to identify groups of residents with similar clinical-demographic characteristics, and a hierarchical cluster analysis was performed using a dissimilarity matrix based on the Gower metric and the complete linkage clustering method. Table 1 reports the clinical and demographic variables that were used for clustering. To assess the robustness of the clustering solution, a bootstrap resampling procedure with 1,000 iterations was implemented. The stability of the resulting solutions was quantified using the adjusted Rand index (ARI) between each resampled clustering and the original solution, and the mean ARI was calculated as an overall measure of cluster reproducibility. After clustering, the characteristics of the resulting groups were examined through descriptive statistics and the incidence of each type of HAI was calculated for each group by means of the following measures: (i) number of each type of HAI divided by the total number of HAIs in the group multiplied by 100; (ii) number of each type of HAI divided by the total number of residents in the group multiplied by 100. In addition, a *t*-test for quantitative variables and a *χ* ^2^ test for qualitative variables were performed to test for any significant differences between the groups. In this study, clustering was applied as a data-driven stratification tool to group residents into distinct multidimensional susceptibility profiles rather than to rank residents by overall frailty; comparisons with a Frailty Index^22^ and the Charlson Comorbidity Index^21^ are reported in Appendix A1.

**Table 1.** Demographic, functional, clinical and comorbidity variables included in the machine-learning clustering analysis Table 1 long description.

**Demographic characteristics**	
	Sex
	Age
**Clinical characteristics**	
	Disorientation
	Mobility
	Incontinence
	Urinary catheter
	Vascular catheter
*Charlson Comorbidity Index conditions* ^1^	
	Myocardial infarction
	Decompensatio cordis
	Peripheral arterial disease
	Cardiovascular disease
	Dementia
	Chronic lung disease
	Peptic ulcer
	Liver disorder
	Hemiplegia
	Kidney disorder
	Chronic urinary disorder
	Malignancies
	Leukemia
	Lymphoma
	Metastases
	Systemic disease^2^
	Diabetes

1 [21].

2 Systemic lupus erythematosus, polymyositis, mixed connective tissue disease, polymyalgia rheumatica, or moderate-to-severe rheumatoid arthritis.

## Results

### Clinical-demographic characteristics and incidence of HAIs in the resident population

The 395 residents in the study population were predominantly women (70%) and had a mean age of 84.6 years (SD 3.1). Their clinical characteristics were heterogenous, with multiple health conditions (mean CCI = 2.9, SD: 2.2) (Table 2).

**Table 2.** Demographic and clinical characteristics of LTCF residents, overall and by groups, after cluster analysis Table 2 long description.

	Total sample		G1		G2		G1 vs. G2 (p-values)^1^
Residents (N)	395		156		239		
**Demographic and clinical characteristics**							
*Sex*							
Female	278	70.4%	100	64.1%	178	74.5%	**
Age, years (mean, SD)	84.6	3.1	83.4	8.9	85.4	10.6	*
*Disorientation*							
Mild	106	26.8%	37	23.7%	69	28.9%	
Moderate	65	16.5%	12	7.7%	53	22.2%	***
Severe	77	19.5%	13	8.3%	64	26.8%	***
*Mobility*							
Ambulant	131	33.2%	108	69.2%	23	9.6%	***
Bedridden	14	3.5%	3	1.9%	11	4.6%	
Wheelchair	250	63.3%	45	28.6%	205	85.8%	***
Incontinence	279	70.6	57	36.5%	222	92.3%	***
Urinary catheter	24	6.1%	8	5.1%	16	6.7%	
Vascular catheter	7	1.8%	2	1.3%	5	2.1%	
Myocardial infarction	30	7.6%	12	7.7%	18	7.5%	
Decompensatio cordis	73	18.5%	28	18.0%	45	18.8%	
Peripheral arterial disease	52	13.2%	18	11.5%	34	14.2%	
Cardiovascular disease	135	34.2%	47	30.1%	88	36.8%	
Dementia	165	41.8%	34	21.8%	131	54.8%	***
Chronic lung disease	64	16.2%	12	7.7%	52	21.8%	***
Peptic ulcer	11	2.8%	5	3.2%	6	2.5%	
Liver disorder	14	3.5%	6	3.8%	8	3.3%	
Hemiplegia	25	6.3%	4	2.6%	21	8.8%	**
Kidney disorder	38	9.6%	7	4.5%	31	13.0%	***
Chronic urinary disorder	60	15.2%	22	14.1%	38	15.1%	
Malignancies	34	8.6%	8	5.1%	26	10.9%	**
Leukemia	3	0.7%	3	1.9%	0	0.0%	
Lymphoma	7	1.8%	3	1.9%		1.7%	
Metastases	7	1.8%	1	0.6%	6	2.5%	
Systemic disease	42	10.6%	30	19.2%	12	5.0%	***
Diabetes	92	23.3%	22	14.1%	70	9.3%	***
Charlson’s index, CCI (mean, SD)	2.9	2.9	2.9	3.0	3.3	3.1	

1 P-values are reported as follows: * .01 ≤ *P* < .05; ** .001 ≤ *P* < .01; *** *P* < .001.

G1 and G2: groups resulting from the machine-learning clustering analysis.

Data source: Italian H4LS data set, 2023.

The total number of HAIs recorded during the study period was 296, with RTIs being the most reported (29.5%, 95% CI 24.2%–31.1%), followed by COVID-19 infections (26.3%, 95% CI 22.1%–28.4%) and UTIs (15%, 95% CI 11.0%–35.4%). Table 3 shows crude and estimated incidence measures by type of HAI.

**Table 3.** Crude percentage, estimated ratio and rate by type of HAIs in the total sample (n = 395) Table 3 long description.

HAIs	N	Crude %	Estimated %	Crude ratio	Estimated ratio (95% CI)	Crude rate	Estimated rate (95% CI)
Total infections	296	100	–	74.9	74.9 (70.3–78.3)	0.23	0.57 (0.11–0.76)
RTIs	93	31.4	29.5 (24.2–31.1)	24.5	24.5 (22.2–27.0)	0.07	0.94 (1.01–1.33)
COVID-19	83	28.0	26.3 (22.1–28.4)	22.7	22.7 (21.2–24.3)	0.07	0.55 (0.39–0.79)
UTIs	48	16.2	15.0 (11.0–35.4)	35.6	35.6 (33.2–38.2)	0.04	0.76 (0.60–0.95)
Gastrointestinal	22	7.4	7.6 (4.8–8.9)	7.8	7.8 (6.7–9.1)	0.02	0.23 (0.13–0.39)
Skin and soft tissues	21	7.1	6.9 (4.4–11.8)	8.7	8.7 (7.6–9.8)	0.02	0.59 (0.39–0.88)
Eye, ear, nose and mouth	17	5.7	5.7 (3.6–6.1)	3.6	3.6 (2.9–4.5)	0.01	0.22 (0.15–0.32)
Other	7	2.4	2.4 (1.1–20.2)	0.5	0.5 (0.3–0.8)	0.01	0.11 (0.08–0.16)
Surgical site	1	0.3	0.4 (0.1–10.7)	19.2	19.2 (17.2–21.4)	0.00	0.01 (0.01–0.03)
Unexplained febrile case	4	1.4	1.4 (1.1–4.9)	0.6	0.6 (0.3–0.9)	0.00	0.04 (0.02–0.07)
Bloodstream	0	0.0	–				

RTIs, respiratory tract infections; UTIs, urinary tract infections.

Data source: Italian H4LS data set, 2023.

### Clustering of residents

The algorithm produced two significant groups, characterized by low intra-group heterogeneity and high inter-group heterogeneity, reflecting the different clinical case mix of the residents. The quality of the clustering solution was good (silhouette coefficient: 0.59) and reproducibility of clusters was high (ARI: 0.83). Group 1 (G1) included 156 residents (39% of the sample); Group 2 (G2) included 239 residents (61%) (Table 2).

Compared to G1, residents in G2 were predominantly women (G1: 64% vs G2: 75%, *P* = .01), disoriented (G1: 40% vs G2: 78%, *P* < .0001), in a wheelchair (G1: 29% vs G2: 86%, *P* < .0001) and incontinent (G1: 37% vs G2: 92%, *P* < .0001). Moreover, regarding the clinical characteristics, G2 included a higher percentage of residents with dementia (G1: 22% vs G2: 55%, *P* = .001), chronic lung disease (G1: 8% vs G2: 22%, *P* < .0001), hemiplegia (G1: 3% vs G2: 9%, *P* = .02), kidney disorders (G1: 7% vs G2: 31%, *P* < .0001), malignancies (G1: 5% vs G2: 11%, *P* = .03) and diabetes (G1: 14% vs G2: 29%, *P* < .0001). In contrast to G2, most of the G1 residents were ambulant (69.2%). G1 was characterized by a significantly higher percentage of residents with systemic disease (G1: 19.2% vs G2: 5.0%, *P* = .001). The mean age of G1 and G2 was 83 (SD 8.9) and 85 (SD 10.6), respectively.

The proportion of HAIs in G2 was significantly higher than in G1 (G1: 65.4% vs. G2: 81.2%, *P* < .0001), highlighting an increased susceptibility of this group to HAIs, including UTIs (G1: 9.8% vs. G2: 19%, *P* < .0001), RTIs (G1: 27.5% vs. G2: 33.5%, *P* = .04) and skin and soft tissues infections (G1: 3.9% vs. G2: 8.8%, *P* < .0001). G1 was characterized by a higher percentage of COVID-19 infections (G1: 43.1% vs. G2: 20.1%, *P* = .02) (Table 4).

**Table 4.** Percentage of type of HAIs by groups Table 4 long description.

HAIs		G1	G2	G1 vs G2 (p-values)^1^
		(n = 156)	(n = 239)	
Total infections	N	102	194	
	% on residents	65.4	81.2	***
UTIs	N	10	38	
	% on HAIs	9.8	19.6	***
	% on residents	6.6	8.0	***
RTIs	N	28	65	
	% on HAIs	27.5	33.5	*
	% on residents	18.5	13.7	***
COVID-19	N	44	39	
	% on HAIs	43.1	20.1	**
	% on residents	29.1	10.3	
Surgical site	N	1	0	
	% on HAIs	1.0	0.0	
	% on residents	0.7	0.0	
Skin and soft tissues	N	4	17	
	% on HAIs	3.9	8.8	***
	% on residents	2.6	3.6	.
Gastrointestinal tract	N	6	16	
	% on HAIs	5.9	8.2	
	% on residents	4.0	3.4	
Eye, ear, nose mouth	N	5	12	
	% on HAIs	4.9	6.2	
	% on residents	3.3	2.5	
Bloodstream	N	0	0	
	% on HAIs	0	0	
	% on residents	0	0	
Unexplained febrile case	N	2	2	
	% on HAIs	2.0	1.0	
	% on residents	1.3	0.4	
Other	N	2	5	
	% on HAIs	2.0	2.6	
	% on residents	1.3	1.1	

RTIs, respiratory tract infections; UTIs, urinary tract infections; HAIs, healthcare-associated infections.

1 *P*-values are reported as follows: .10 < *P* < .05; * .01 ≤ *P* < .05; ** .001 ≤ *P* < .01; *** *P* < .001.

Data source: Italian H4LS data set, 2023.

**Figure 1.**
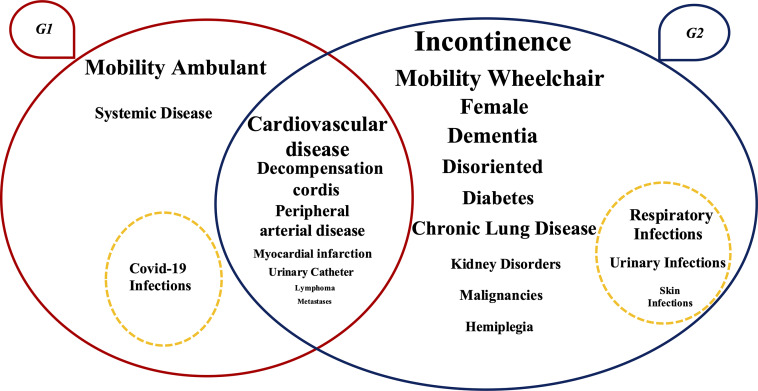
Figure 1 long description. Visual representation of the clustering results (N = 395). *Note*: G1 and G2 are represented by ovals with the corresponding clinical conditions reported within them. The conditions positioned at the intersection of the ovals are those that do not show statistically significant differences between the two groups. Conversely, the conditions placed within each oval are those that differ significantly between the groups; each condition is reported in the group where it has a higher prevalence. The font size is proportional to the percentage of residents presenting each clinical condition. The dashed circle indicates the HAIs that are more prevalent in each group.

Figure 1 summarizes the clustering results shown in Tables 2 and 4.

## Discussion

The longitudinal design of the study provides important evidence on the incidence of HAIs in a relatively large sample of Italian LTCFs. The estimated overall HAI incidence of 0.6 per 1,000 resident days complements incidence rates from earlier European studies, underscoring the need for targeted surveillance and prevention strategies.^5,6^

Beyond its epidemiological contribution, this study extends the application of AI, particularly ML methods, from their predominant use in acute-care hospital settings,^13–17^ to LTCFs, suggesting their potential to predict HAIs and inform antimicrobial stewardship in this context as well.

Rather than relying solely on frailty, this method identified a highly stable two-cluster stratification, that captured multidimensional clinical profiles reflecting underlying health status and differential susceptibility to specific types of HAIs. The observed high level of cluster reproducibility indicates that the ML-derived resident stratification was robust and unlikely to result from sampling variability. Such stability reinforces the value of an ML-based approach for reliably defining HAI susceptibility profiles in LTCFs and supports its potential use as an early-warning component for IPC. In addition, the identification of high-risk clusters may enhance antimicrobial stewardship by prioritizing targeted screening and guiding differentiated antibiotic protocols, thereby reducing inappropriate antimicrobial use.

Compared to Group 1, Group 2 included more clinically vulnerable residents with higher exposure to RTIs, UTIs, and skin and soft tissues infections. These infections were significantly more frequent among residents with a high prevalence of underlying health conditions such as disorientation, incontinence, low mobility, hemiplegia, kidney disorder, dementia, malignancies, diabetes, and chronic lung disease. Most of these conditions are well-recognized risk factors for bacterial HAIs, particularly UTIs and skin or soft-tissue infections. The observed co-occurrence of incontinence and UTIs, and of chronic lung disease and RTIs aligns with previous reports and supports the biological plausibility of these findings.^3,4,23,24^ In contrast, Group 1 residents, characterized by better mobility, greater functional autonomy, preserved cognitive status, and by a lower overall comorbidity burden, showed a higher proportion of COVID-19 infections. Greater mobility within the facility and increased interpersonal contact may have facilitated exposure to respiratory viral pathogens, consistent with patterns described in LTCF COVID-19 outbreaks.^25^ Notably, G1 included residents with clinically significant systemic diseases (as defined in Table 2), underscoring that serious inflammatory conditions may occur despite a relatively low overall comorbidity burden, reflecting substantial heterogeneity across individuals and among different rheumatic disorders.^26^

Prior studies have demonstrated that variation in resident frailty, disability, and clinical case mix substantially influences infection risk, prompting efforts to operationalize these differences using administrative measures such as the Case-Mix Index (CMI) and Resource Utilization Groups.^27–30^ However, no consistent association was observed between CMI and infection rates, with the notable exception of a study conducted in the same Italian regions included in the present analysis.^31^ Taken together, these findings suggest that traditional administrative measures may not fully capture the multidimensional clinical vulnerability underlying susceptibility to HAIs in LTCFs. Likewise, frailty indices and comorbidity scores summarize vulnerability primarily as an overall burden of deficits, limiting their ability to differentiate distinct clinical profiles within already frail LTCF populations.^32,33^ In contrast, the unsupervised clustering approach adopted in this study offers a methodologically distinct and complementary perspective: rather than ranking residents along a single continuum of frailty or comorbidity, it integrates functional, cognitive, clinical, and care-related variables simultaneously, without imposing predefined weights or linear assumptions. This data-driven framework may facilitate the identification of latent clinical profiles defined by specific combinations of characteristics, thereby helping to capture heterogeneity that could be masked by unidimensional summary measures.

Consistent with this perspective, the two clusters identified in this study share several underlying health conditions, reflecting the generally high baseline vulnerability of the LTCF population. However, they differ in functional, cognitive, continence, and care-dependency patterns that may better capture infection susceptibility than overall frailty.

This approach aligns with contemporary models of geriatric care, which move beyond the traditional view of LTCF populations as uniformly frail, and instead adopt a more dynamic, risk-adjusted perspective.^11,32^ In this context, the integration of ML techniques into epidemiological surveillance enhances early warning capabilities and supports evidence-based clinical decision-making through resident-level data stratification. The well-documented severe consequences of HAIs among LTCF residents reinforce the urgency of identifying high-risk resident groups and tailoring preventive interventions accordingly.^34^ Additionally, high rates of colonization with multidrug-resistant organisms, as observed in Italian LTCFs,^35^ further underscore the critical need for proactive surveillance and stratification strategies to mitigate infection risk.

Some limitations of this study should be considered when interpreting the findings. Incomplete resident enrollment due to informed consent requirements prevented a full census in some facilities, potentially limiting representativeness. Such barriers to comprehensive surveillance are well recognized in LTCFs, where resident turnover and consent procedures often hinder systematic data collection.^4,36^ Generalisability may be further constrained by the heterogeneity of participating LTCFs and their differing case-mix profiles, as well as by the use of a convenience sample, as previously noted in the H4LS study.^37^ Reliance on manual data collection rather than routinely collected electronic data in LTCFs constrains the scalability of AI-based approaches. This highlights the need for shared data repositories that integrate resident-level information across facilities. Access to such data would enable the application of information extraction methods such as clustering, to classify facilities based on the proportion of residents at high risk for HAIs. Finally, although formal statistical testing of associations between comorbidities and HAIs was not feasible with the present data set, and the observed cluster-specific infection profiles should therefore be considered as hypothesis-generating rather than confirmatory, these patterns suggest that preventive strategies may benefit from being tailored to residents’ functional and clinical profiles.

In conclusion, this study indicates that AI-based clustering is feasible in LTCFs and may complement traditional infection risk assessment methods. In a field where AI applications remain limited, these findings support further investigation of machine-learning approaches using larger and more comprehensive data sets. By capturing infection-specific risk profiles that extend beyond global frailty, ML-based clustering highlights subgroups with higher HAI incidence and may inform targeted prevention and more efficient resource allocation. Future research should assess the reproducibility of these results across diverse LTCF settings and evaluate the impact of AI-driven risk stratification on resident outcomes, healthcare costs, and infection prevention strategies.

## Supporting information

Leucci et al. supplementary material Leucci et al. supplementary material

## Data Availability

Pseudonymised case-based data or aggregate data are available upon a data request for research purposes to the ECDC (https://www.ecdc.europa.eu/en/publications-data/request-tessy-data-research). Following the approval of the data request, the relevant data can be shared through a secure online platform. The data can be made available for a minimum of 10 years from the end of the study.

## Supplementary material

The supplementary material for this article can be found at https://doi.org/10.1017/ice.2026.10413.

## Figure 1 Long description

A Venn diagram illustrates the clustering results of clinical conditions in two groups of residents in long-term care facilities. The diagram consists of two overlapping ovals labeled G1 and G2. The oval labeled G1 represents the group with conditions such as systemic disease, cardiovascular disease, decompensation cordis, peripheral arterial disease, myocardial infarction, urinary catheter, lymphoma, metastases, and COVID-19 infections. The oval labeled G2 represents the group with conditions such as incontinence, mobility wheelchair, female, dementia, disoriented, diabetes, chronic lung disease, respiratory infections, urinary infections, skin infections, kidney disorders, malignancies, and hemiplegia. The overlapping section between G1 and G2 includes cardiovascular disease, indicating that this condition does not show statistically significant differences between the two groups. Conditions within each oval are those that differ significantly between the groups, with each condition reported in the group where it has a higher prevalence. The font size of each condition is proportional to the percentage of residents presenting that clinical condition. A dashed circle within G1 indicates that COVID-19 infections are more prevalent in this group, while a dashed circle within G2 indicates that respiratory infections, urinary infections, and skin infections are more prevalent in this group.

Navigate back to Figure 1.

## Table 1 Long description

The table presents demographic, functional, clinical, and comorbidity variables included in a machine-learning clustering analysis. It is divided into three main sections: Demographic characteristics, Clinical characteristics, and Charlson Comorbidity Index conditions. The Demographic characteristics section includes Sex and Age. The Clinical characteristics section lists Disorientation, Mobility, Incontinence, Urinary catheter, and Vascular catheter. The Charlson Comorbidity Index conditions section includes Myocardial infarction, Decompensatio cordis, Peripheral arterial disease, Cardiovascular disease, Dementia, Chronic lung disease, Peptic ulcer, Liver disorder, Hemiplegia, Kidney disorder, Chronic urinary disorder, Malignancies, Leukemia, Lymphoma, Metastases, Systemic disease, and Diabetes. The table has three columns and twenty rows, providing a comprehensive list of variables used for clustering residents with similar clinical-demographic characteristics.

Navigate back to Table 1.

## Table 2 Long description

The table presents demographic and clinical characteristics of 395 long-term care facility residents, divided into two groups, G1 and G2. It includes data on sex, age, disorientation levels, mobility, incontinence, and various health conditions. The table has 31 rows and 5 columns, with columns for total sample, G1, G2, and p-values comparing G1 and G2. Notable trends include a higher percentage of females in G2 (74.5%) compared to G1 (64.1%), and a higher mean age in G2 (85.4 years) than in G1 (83.4 years). Disorientation levels vary significantly, with severe disorientation more prevalent in G2 (26.8%) than in G1 (8.3%). Mobility differences are stark, with 69.2% of G1 residents being ambulant compared to only 9.6% in G2. Incontinence is more common in G2 (92.3%) than in G1 (36.5%). Health conditions such as dementia, chronic lung disease, and kidney disorders show significant differences between the groups. The Charlson's Comorbidity Index (CCI) is slightly higher in G2 (3.0) compared to G1 (2.9).

Navigate back to Table 2.

## Table 3 Long description

The table presents crude and estimated incidence measures by type of healthcare-associated infections (HAIs) in a total sample of 395 individuals. It includes columns for the number of cases, crude percentage, estimated percentage with confidence intervals, crude ratio, estimated ratio with confidence intervals, crude rate, and estimated rate with confidence intervals. The table lists various types of HAIs such as respiratory tract infections (RTIs), COVID-19, urinary tract infections (UTIs), gastrointestinal infections, skin and soft tissue infections, eye, ear, nose, and mouth infections, other infections, surgical site infections, unexplained febrile cases, and bloodstream infections. RTIs are the most reported, followed by COVID-19 infections and UTIs. Each row provides detailed data on the incidence measures for each type of HAI.

Navigate back to Table 3.

## Table 4 Long description

The table compares the percentage of healthcare-associated infections (HAIs) between two groups, G1 and G2. It includes data on total infections, urinary tract infections (UTIs), respiratory tract infections (RTIs), COVID-19, surgical site infections, skin and soft tissue infections, gastrointestinal tract infections, eye, ear, nose, and mouth infections, bloodstream infections, unexplained febrile cases, and other infections. The table has 12 rows and 5 columns, with column headers including HAIs, G1, G2, and G1 vs G2 (p-values). Notable trends include a higher proportion of HAIs in G2 compared to G1, with significant differences in UTIs, RTIs, and skin and soft tissue infections. G1 has a higher percentage of COVID-19 infections. The data highlights increased susceptibility to HAIs in G2.

Navigate back to Table 4.

## References

[1] European Centre for Disease Prevention and Control, Latour K , Kärki T. Point prevalence survey of healthcare-associated infections and antimicrobial use in European long-term care facilities: 2016–2017. Stockholm: ECDC;2023. 10.2807/1560-7917.ES.2018.23.46.1800394 PMC6247460 30458913

[2] Cristina ML , Spagnolo AM , Giribone L , Demartini A , Sartini M. Epidemiology and prevention of healthcare-associated infections in geriatric patients: a narrative review. Int J Environ Res Public Health 2021;18:5335. doi: 10.3390/ijerph18105335. 34067797 PMC8156303

[3] Bennett N , Tanamas SK , James R , et al. Healthcare-associated infections in long-term care facilities: a systematic review and meta-analysis of point prevalence studies. BMJ Public Health 2024;2:e000504. doi: 10.1136/bmjph-2023-000504. 40018192 PMC11816188

[4] European Centre for Disease Prevention and Control. Point prevalence survey of healthcare-associated infections and antimicrobial use in European long-term care facilities. Stockholm: ECDC; 2025.

[5] Engelhart ST , Hanses-Derendorf L , Exner M , Kramer MH. Prospective surveillance for healthcare-associated infections in German nursing home residents. J Hosp Infect 2005;60:46–50. doi: 10.1016/j.jhin.2004.09.027. 15823656

[6] König E , Medwed M , Pux C , et al. Prospective surveillance of healthcare-associated infections in residents in four long-term care facilities in Graz, Austria. Antibiotics (Basel) 2021;10:509. doi: 10.3390/antibiotics10050509. 34067175 PMC8151996

[7] Vicentini C , Russotto A , Bazzolo S , et al. Implementation of a centralized, web-based surveillance for healthcare-associated infections among residents of long-term care facilities in Italy. Public Health Pract (Oxf) 2023;6:100421. doi: 10.1016/j.puhip.2023.100421. 37661965 PMC10472289

[8] Bordino V , Vicentini C , D’Ambrosio A , Quattrocolo F , Zotti CM. Burden of healthcare-associated infections in Italy: incidence, attributable mortality and disability-adjusted life years (DALYs) from a nationwide study, 2016. J Hosp Infect 2021;113:164–171. doi: 10.1016/j.jhin.2021.04.008. 33940090

[9] Matheï C , Niclaes L , Suetens C , Jans B , Buntinx F. Infections in residents of nursing homes. Infect Dis Clin North Am 2007;21:761–772. doi: 10.1016/j.idc.2007.07.005. 17826622

[10] Ricchizzi E , Sasdelli E , Leucci AC , et al. Incidence of healthcare-associated infections in long-term care facilities: a 12-month longitudinal study across European countries. Lancet Infect Dis 2025;25:1199–1207. doi: 10.1016/S1473-3099(25).

[11] Fazio S , Pace D , Flinner J , Kallmyer B. The fundamentals of person-centered care for individuals with dementia. Gerontologist 2018;58:S10–S19. doi: 10.1093/geront/gnx122. 29361064

[12] Sverdrup K , Bergh S , Selbæk G , Røen I , Kirkevold Ø. , Tangen GG. Mobility and cognition at admission to the nursing home: a cross-sectional study. BMC Geriatr 2018;18:30. doi: 10.1186/s12877-018-0719-4. 29378518 PMC5789666

[13] Wang J , Wang G , Wang Y , Wang Y. Development and evaluation of a model for predicting the risk of healthcare-associated infections in patients admitted to intensive care units. Front Public Health 2024;12:1444176. doi: 10.3389/fpubh.2024.1444176. 39329001 PMC11424534

[14] Murri R , De Angelis G , Antenucci L , et al. A machine learning predictive model of bloodstream infection in hospitalized patients. Diagnostics (Basel) 2024;14:445. doi: 10.3390/diagnostics14040445. 38396484 PMC10887662

[15] Chang CW , Chang CH , Chien CY , et al. Predictive modelling of hospital-acquired infection in acute ischemic stroke using machine learning. Sci Rep 2024;14:31066. doi: 10.1038/s41598-024-31066-0. 39730788 PMC11680783

[16] Cho, Y , Lee, HK , Kim, J , et al. Prediction of hospital-acquired influenza using machine learning algorithms: a comparative study. BMC Infect Dis 2024;24:466. doi: 10.1186/s12879-024-09358-1. 38698304 PMC11067145

[17] El Arab RA. Artificial intelligence in hospital infection prevention: an integrative review. J Hosp Infect 2025;137:12–20. doi: 10.1016/j.jhin.2025.01.004. PMC12001280 40241963

[18] Arzilli, G , De Vita, E , Pasquale, M , et al. Innovative techniques for infection control and surveillance in hospital settings and long-term care facilities: a scoping review. Antibiotics (Basel) 2024;13:77. doi: 10.3390/antibiotics13010077. 38247635 PMC10812752

[19] Vandenbroucke, JP , von Elm, E , Altman, DG , et al. Strengthening the reporting of observational studies in epidemiology (STROBE): explanation and elaboration. PLoS Med 2007;4:e297. doi: 10.1371/journal.pmed.0040297. 17941715 PMC2020496

[20] European Centre for Disease Prevention and Control. Protocol for point prevalence surveys of healthcare-associated infections and antimicrobial use in European long-term care facilities – version 4.0. Stockholm: ECDC; 2023.

[21] Buntinx F , Niclaes L , Suetens C , Jans B , Mertens R , Van den Akker M. Evaluation of Charlson’s Comorbidity Index in elderly living in nursing homes. J Clin Epidemiol 2002;55:1144–1147. 12507679 10.1016/s0895-4356(02)00485-7

[22] Searle SD , Mitnitski A , Gahbauer EA , Gill TM , Rockwood K. A standard procedure for creating a frailty index. BMC Geriatr 2008;8:24. doi: 10.1186/1471-2318-8-24. 18826625 PMC2573877

[23] Furmenti MF , Rossello P , Bianco S , et al. Healthcare-associated infections and antimicrobial use in long-term care facilities (HALT3): an overview of the Italian situation. J Hosp Infect 2019;102:425–430. doi: 10.1016/j.jhin.2019.02.007. 30790605

[24] Baranowska-Tateno, K , Micek, A , Gniadek, A , Wójkowska-Mach, J , Różańska, A. Healthcare-associated infections and prevention programs in general nursing versus residential homes—results of the point prevalence survey in Polish long-term care facilities. Medicina 2024;60:137. doi: 10.3390/medicina60010137. 38256397 PMC10820304

[25] McAndrew F , Sacks-Davis R , Abeysuriya RG , Delport D , West D , Parta I , Majumdar S , Hellard M , Scott N . COVID-19 outbreaks in residential aged care facilities: an agent-based modeling study. Front Public Health 2024;12:1344916. doi: 10.3389/fpubh.2024.1344916. 38835609 PMC11148262

[26] Radner H . Multimorbidity and rheumatic conditions-enhancing the concept of comorbidity Nat Rev Rheumatol 2014;10(4):252–6. doi: 10.1038/nrrheum.2013.212. 24418765

[27] Fries BE , Schneider DP , Foley WJ , et al. Refining a case-mix measure for nursing homes: Resource Utilization Groups (RUG-III). Med Care 1994;32:668–685. 8028403 10.1097/00005650-199407000-00002

[28] Mylotte JM , McDermott C , Spooner J. Trends in antibiotic use and cost and influence of case-mix variation in a long-term care facility. Am J Infect Control 2003;31:18–25. 12548253 10.1067/mic.2003.47

[29] Mylotte JM. Antibiotic stewardship in long-term care: metrics and risk adjustment. J Am Med Dir Assoc 2016;17:672.e13–672.e18. 10.1016/j.jamda.2016.04.014 27233489

[30] Mylotte JM. Risk adjustment for benchmarking nursing home infection surveillance data: a narrative review. Am J Infect Control 2021;49:366–374. doi: 10.1016/j.ajic.2020.08.006. 32791257

[31] Marchi M , Grilli E , Mongardi M , Bedosti C , Nobilio L , Moro ML. Prevalence of infections in long-term care facilities: how to read it? Infection 2012;40:493–500. 22576022 10.1007/s15010-012-0266-1

[32] Clegg A , Young J , Iliffe S , Rikkert MO , Rockwood K. Frailty in elderly people. Lancet 2013;381:752–762. 23395245 10.1016/S0140-6736(12)62167-9 PMC4098658

[33] Charlson ME , Pompei P , Ales KL , MacKenzie CR. A new method of classifying prognostic comorbidity in longitudinal studies: development and validation. J Chronic Dis 1987;40:373–383. 3558716 10.1016/0021-9681(87)90171-8

[34] Koch, AM , Eriksen, HM , Elstrøm, P , Aavitsland, P , Harthug, S Severe consequences of healthcare-associated infections among residents of nursing homes: a cohort study. J Hosp Infect 2009;71:269–274. doi: 10.1016/j.jhin.2008.11.019. 19147254

[35] Giufrè, M , Ricchizzi, E , Accogli, M , et al. Colonization by multidrug-resistant organisms in long-term care facilities in Italy: a point-prevalence study. Clin Microbiol Infect 2017;23:961–967. doi: 10.1016/j.cmi.2017.04.015. 28412380

[36] Juthani-Mehta M , Quagliarello VJ. Infectious diseases in the nursing home setting: challenges and opportunities for clinical investigation. Clin Infect Dis 2010;51:931–936. doi: 10.1086/656411. 20822459 PMC3083824

[37] Kohler P , McGeer A. Infections in long-term care: burden affirmed, action needed. Lancet Infect Dis 2025;25:1163–1164.

