# Supplementary material for: Healthcare-associated infections in Italian long-term care facilities: a machine learning analysis of a 12-month cohort
Source: Infect Control Hosp Epidemiol. 2026 Apr 8;47(6):578–85. doi: 10.1017/ice.2026.10413 (PMC13216815; doi:10.1017/ice.2026.10413)
Supplement: Leucci et al. supplementary material [file S0899823X26104139sup001.docx]

**APPENDIX**

**A1. Comparative analysis with a Frailty Index and Charlson Index**

This comparative analysis was designed to evaluate whether ML-based clustering provides information beyond that captured by a traditional unidimensional frailty score.

A frailty index (FI) was constructed following the deficit accumulation model originally proposed by Searle et al.^22^ Briefly, individual health deficits were coded as binary variables and summed up to generate a continuous index reflecting the proportion of deficits present in each resident.

The FI included 22 deficits: 17 chronic conditions derived from the Charlson Comorbidity Index (CCI) ^21^ and 5 functional and clinical variables routinely collected at baseline (Table 1)

Each deficit was coded as 0 (absence) or 1 (presence), and the FI was calculated as the ratio between the number of deficits present and the total number of deficits considered. Higher FI values indicated greater frailty.

In addition to the FI, the CCI was also evaluated as an independent comparator, in order to disentangle the role of comorbidity burden from multidimensional frailty and ML-derived resident profiles.

To compare the predictive performance of the frailty-based and ML-based approaches, we fitted GEE Poisson regression models for four major infection sites (i) urinary tract infections (UTIs), (ii) respiratory tract infections (RTIs), (iii) COVID-19, and (iv) skin and soft tissue infections.

For each infection site, tree models were estimated: (a) a model including cluster membership (G2 vs G1) as the main exposure, (b) a model including the frailty index as the main exposure and (iii) a model including the CCI as the main exposure

All models were adjusted for age and sex and accounted for within-facility correlation using GEE. Results are reported as incidence rate ratios (IRRs) with 95% confidence intervals.

Figure A1 presents the results of the comparative analysis between the FI, CCI and the ML-based clustering approach, reported as incidence rate ratios (IRRs) derived from GEE models. For UTIs, both the frailty index and cluster membership were similarly associated with infection incidence (FI: IRR 1.78, 95% CI 1.41–2.24; Cluster G2 vs G1: IRR 1.72, 95% CI 1.37–2.16), while the CCI showed a weaker association (CCI:IRR 1.15, 95%IC 1.07-1.25).

Comparable results were observed for respiratory tract infections (RTIs), with overlapping effect estimates for the frailty index (IRR 1.40, 95% CI 1.17–1.67) and cluster membership (IRR 1.40, 95% CI 1.11–1.68), again exceeding the discriminatory capacity of the CCI alone (CCI: IRR 1.25, 95%IC 1.15-1.40)

For COVID-19, the FI an CCI were not associated with infection incidence (FI:IRR 0.93, 95% CI 0.75–1.16; CCI:IRR 0.98, 95%IC 0.89-1.07), whereas cluster membership was significantly associated with a lower incidence in Group 2 (IRR 0.55, 95% CI 0.37–0.79), indicating higher COVID-19 incidence among residents in Group 1.

Similarly, for skin and soft tissue infections, the FI and CH were not significantly associated with infection incidence (FI: IRR 1.32, 95% CI 0.89–1.96;CCI:IRR 1.11,95% CI 0.93-1.38), while cluster membership showed a significant association, with higher incidence in Group 2 (IRR 1.46, 95% CI 1.33–1.60).

Overall, these results indicate that frailty and ML-derived clusters provide comparable information for UTIs and RTIs, while cluster membership better discriminates infection risk for COVID-19 and skin and soft tissue infections.

**Legend for Figure A1**

**[Figure A1.** Incidence rate ratios (IRRs) comparing frailty index–based, Charlson Comorbidity Index and ML-derived cluster approaches across infection sites]

[NOTE of Figure A1:

**Note:**
*Incidence rate ratios (IRRs) and 95% confidence intervals from generalized estimating equation (GEE) Poisson models for four infection sites (urinary tract infections, respiratory tract infections, COVID-19, and skin and soft tissue infections). For each site, results from models including the frailty index, Charlson Comorbidity Index or ML-derived cluster membership (Group 2 vs Group 1) as the main exposure are shown. All models were adjusted for age and sex.]*
